# Quantification of Microvascular Lesions in the Central Retinal Field: Could It Predict the Severity of Diabetic Retinopathy?

**DOI:** 10.3390/jcm12123948

**Published:** 2023-06-09

**Authors:** Jimena Fernández-Carneado, Ana Almazán-Moga, Dolores T. Ramírez-Lamelas, Cristina Cuscó, José Ignacio Alonso de la Fuente, J. Carlos Pastor, María Isabel López Gálvez, Berta Ponsati

**Affiliations:** 1 BCN Peptides, S.A., Polígon Industrial Els Vinyets-Els Fogars II, 08777 Sant Quintí de Mediona, Barcelona, Spain; jfernandez@bcnpeptides.com (J.F.-C.); aalmazan@bcnpeptides.com (A.A.-M.); drlamelas@live.com (D.T.R.-L.); ccusco@bcnpeptides.com (C.C.); 2 IOBA Reading Center, University of Valladolid, Paseo de Belén, 17, 47011 Valladolid, Spain; jialfue@gmail.com (J.I.A.d.l.F.); maribel@ioba.med.uva.es (M.I.L.G.)

**Keywords:** retinopathy screening, microvascular complications, fundoscopy, clinical utility, clinical research

## Abstract

Diabetic retinopathy (DR) is a neurodegenerative disease characterized by the presence of microcirculatory lesions. Among them, microaneurysms (MAs) are the first observable hallmark of early ophthalmological changes. The present work aims to study whether the quantification of MAs, hemorrhages (Hmas) and hard exudates (HEs) in the central retinal field could have a predictive value on DR severity. These retinal lesions were quantified in a single field NM-1 of 160 retinographies of diabetic patients from the IOBA’s reading center. Samples included different disease severity levels and excluded proliferating forms: no DR (*n* = 30), mild non-proliferative (*n* = 30), moderate (*n* = 50) and severe (*n* = 50). Quantification of MAs, Hmas, and HEs revealed an increasing trend as DR severity progresses. Differences between severity levels were statistically significant, suggesting that the analysis of the central field provides valuable information on severity level and could be used as a clinical tool to assess DR grading in the eyecare routine. Even though further validation is needed, counting microvascular lesions in a single retinal field can be proposed as a rapid screening system to classify DR patients with different stages of severity according to the international classification.

## 1. Introduction

### 1.1. Diabetic Retinopathy

A total of 537 million people worldwide were estimated to have diabetes mellitus (DM) in 2021, representing 10.5% of the global adult population (20–79 years). This number is expected to increase to 643 million (11.3%) by 2030 and up to 783 million (12.2%) by 2045 [1].

A very frequent complication of DM is diabetic retinopathy (DR), a classically considered microcirculatory disease, but currently defined also as a neuropathy [2,3]. DR has become the leading cause of blindness in working-age adults (20–65 years) in the most developed countries. Sustained hyperglycemia is considered to play a crucial role in the pathogenesis of retinal microvascular damage, so the monitoring of blood glucose and glycated hemoglobin (HbA1c) levels [4] has substantially contributed to arrest DR progression. However, the high prevalence of visual impairment and blindness has increased since 1990, mainly due to type II diabetes or in other words as a consequence of a growing and ageing diabetic population [5].

Chronic hyperglycemia induces a series of biochemical pathways in glucose metabolism, rheological changes in blood flow and anatomical abnormalities in the vascular wall that trigger the microangiopathy in arterioles, capillaries and vessels. Multiple metabolic mechanisms have been implicated the physiopathology of diabetic retinopathy, such as the polyol pathway, advanced end products (AGEs) accumulation, the protein kinase C (PKC) pathway, inflammation, vascular endothelial growth factor (VEGF) expression, the hexosamine pathway, renin-angiotensin upregulation and oxidative stress [4,6].

The earliest responses of retinal blood vessels to this microangiopathy are vessel dilatation and blood flow changes, which are autoregulation mechanisms to increase metabolism in diabetic subjects. Loss of pericytes, that provide structural support to capillaries, is another consequence of uncontrolled glucose concentration, which leads to an increased permeability and localized outpouching of capillary walls (formation of microaneurysms). These processes can further progress to the apoptosis of endothelial cells, thickening of the basement membrane and even capillary occlusion and retinal ischemia, activating the VEGF pathways to stimulate the formation of new blood vessels, that compensate the lack of oxygen and nutrients supply (neovascularization, NV) [7,8].

NV is actually the key pathophysiological process that determines if a patient has entered the proliferative stage, which is known as proliferative diabetic retinopathy (PDR). Before that, when there is no evidence of new blood vessel growth, the patient remains as non-proliferative DR (NPDR) [9]. DR is an asymptomatic disease until the central retinal area, responsible for the sharp vision, becomes affected at severe stages (late NPDR or PDR) [10,11].

The NPDR stage is further characterized by the presence of different types of retinal vascular lesions, such as microaneurysms (MAs), hemorrhages (Hmas), hard exudates (HEs), cotton-wool spots (CWS), intraretinal microvascular abnormalities (IRMA) and venous beading (BV), among others [12]. The combination of these retinal lesions and their relative presence define the specific severity of an NPDR patient. Despite such a wide range of retinal lesions, the earliest clinical sign of DR are MAs, small dilations of the capillaries as a consequence of the loss of pericytes, apoptosis of endothelial cells accompanied of thickening of the basement membrane and capillary occlusion [4]. MAs are clinically identified as small red dots, with increased permeability, located in the small retinal vessels [13]. They are known to be highly dynamic lesions, meaning that, over the course of the disease, some of them may disappear as a consequence of spontaneous occlusion and progressive remodeling of retinal vasculature [14]. 

MAs may bleed and result in Hmas, which can also appear independently, in different shapes and sizes depending on their location. Vascular damage can also lead to the leakage of fluid and lipoproteins into the outer plexiform layer, forming intraretinal edema and the so-called HEs. They are irregularly shaped yellow-white spots that can coalesce with each other forming streaks or clusters most of them centered by microvascular leaking structures [12].

The appearance of MAs is a hallmark of early changes in the retina, so they are considered the first pathological clinical sign for ophthalmoscopic DR diagnosis [15,16]. Analysis of MAs has been a major focus of interest since different studies have demonstrated that their presence, closely centered on the macula, may be associated with complications that cause visual impairment by worsening DR or diabetic macular edema (DME) and onset of PDR [17]. In fact, several studies have demonstrated that the total number of MAs as well as MAs turnover rate (MAT) (i.e., MA formation rate plus MA disappearance rate) in the central area of the retina are indicators of disease progression, suggesting that even a single MA may have a predictive value in DR progression [18,19]. In this sense, the *Wisconsin Epidemiologic Study of Diabetic Retinopathy* in 1989 was the first to demonstrate that the number of MAs at baseline plays an important role as predictor of progression of DR [20]. Later, Klein et al. established, in 4- and 10-year follow-up studies of mild NPDR patients with only MAs at baseline, the relationship between higher MAs number and the progression of DR to moderate NPDR which is a clinically relevant severity level from which the risk of subsequent development of PDR or clinically significant macular edema (CSME) is appreciable [21]. Kohner et al. also confirmed that the number of MAs has a highly predictive value for worsening DR in a 12-year follow-up study from the same population [15].

### 1.2. Diabetic Retinopathy Classification

DR is currently classified following different grading protocols depending on the scope of application, but the current validated systems still rely on semi-qualitative methods. On one hand, in the daily clinical practice, DR is classified following the International Clinical Diabetic Retinopathy (ICDR) disease severity level into four severity levels: mild, moderate, severe NPDR and PDR [18], as shown in Table 1 [18,22]. On the other hand, in clinical trials (CT), the stages of DR are further detailed and classified according to the Early Treatment Diabetic Retinopathy Study (ETDRS) severity scale. It allows the evaluation of additional changes within the same ICDR disease severity level, as shown in the same table. For the ETDRS classification, 7-field standard Color Fundus Photography (CFP) stereoscopic images are acquired and graded comparing them to validated standards [23]. 

According to Table 1, DR grading on either of these systems (ICDR and ETDRS) is not based on the quantification of retinal findings, so one level of severity can actually entail a different casuistry of lesions. Therefore, new metrics able to detect slight changes in the DR severity that rely on quantifiable microvascular lesions could be exploited, combined with AI-automated detection and quantification tools for a fast-track DR primary diagnosing and patient follow-up, supporting eye health care systems.

Although the development of renewed methods is still being discussed [10], the ETDRS is currently the gold standard and the approved system for regulatory assessments. However, it is not implemented in the ophthalmological practice due to practical reasons. Firstly, due to the need for the adequate technical equipment and skilled personnel trained to correctly acquire the 7-field CFP images of the retina, which includes the challenges of capturing appropriate images of peripheral fields. Secondly, due to the need for experienced ophthalmologists able to differentiate between sequential severity levels, e.g., patients with moderate DR (ETDRS 35, ETDRS 43 or ETDRS 47 level), and, finally, due to the requirement for patient cooperation in long sessions [1,25].

In 2001, Bursell et al. validated a new protocol to simplify the examination and diagnosis of DR severity, known as the Joslin Vision Network protocol (JVN) [26]. Instead of 7-field CFP, this system uses 3 non mydriatic (NM) 45°-field stereoscopic color images (named NM-1, NM-2 and NM-3). When compared to the use of the 7-fields standard CFP in the screening of DR, the sensitivity and specificity for detecting referable levels of DR were 82% and 92% for the 3-fields (NM-1, NM-2 and NM-3) and 71% and 96%, respectively, for the central field NM-1 alone. This 3-field system was found to be as effective for DR screening and severity classification (in mild, moderate, severe and PDR levels) as the standard 7-fields 30–35 mm fundus photographs, so it was implemented due its advantage in terms of training, time and cost [25].

Further attempts to reduce the number of images needed for determining DR and defining the severity level have been undertaken. In this sense, the approach of two 45° digital CFP is used in the first autonomous artificial intelligence (AI)-based device for automatic diagnosis of RD with FDA approval for its use in clinical practice [27]. Other studies have evaluated the effectiveness of a single central field 45° digital CFP. These studies demonstrated a sensitivity and specificity for DR screening between 70–95% and these have been translated into clinical benefit with the approval of some AI-based DR detection tools [28]. However, although recording only the central field simplifies the procedures, a single image of the retina is not considered representative enough for the definition of the severity level in clinical practice. 

Considering that DR microvascular changes can be diagnosed only by detecting the presence of at least 1 MA in a single CFP image, we hypothesize that findings in the central retinal area could predict with sufficient accuracy the severity levels estimated by the JVN protocol. This has the pathophysiological basis that the central area is the most susceptible to metabolic alterations of DM [29,30].

Overall, our objective is to study the relationship between the number of microvascular lesions (MAs, Hmas and HEs) in the central retinal field (NM-1) of fundus images and the DR severity level in NPDR patients. Although a number of studies show a relationship between the presence of MAs close to the macula and DR progression, this is the first time that a statistically significant association is reported between the quantification of these lesions and the DR severity level.

## 2. Materials and Methods

The CFP images were provided by the Instituto de Oftalmobiología Aplicada (IOBA) of the University of Valladolid (IOBA-UVA) reading center (Valladolid, Spain) from their image data set. IOBA-UVA reading center possesses a CFP database, from the DR blindness prevention program of the Junta de Castilla y León (Regional Government), with images captured and read by 2 certified readers using the JVN system. This is a retrospective study in which all subjects signed an informed consent for transferring their data and images for teaching and research purposes. Moreover, this research was carried out under the full compliance of the General Data Protection Regulation (GDPR). 

Images were captured using a Topcon TRC-NW400 automatic retinal camera (Topcon Medical Systems, Inc., Oakland, NJ, USA). Three retinal fields at a 45-degree field were acquired according to the JVN grading protocol: NM-1 (centered between the disc and macula), NM-2 (superotemporal vascular arcades) and NM-3 (inferonasal retina). The acquisition protocol was performed under mydriasis with tropicamide. After recording the fundus images, the photographs were classified following the ICDR system: no DR, mild NPDR, moderate NPDR and severe NPDR.

**Figure 1:**
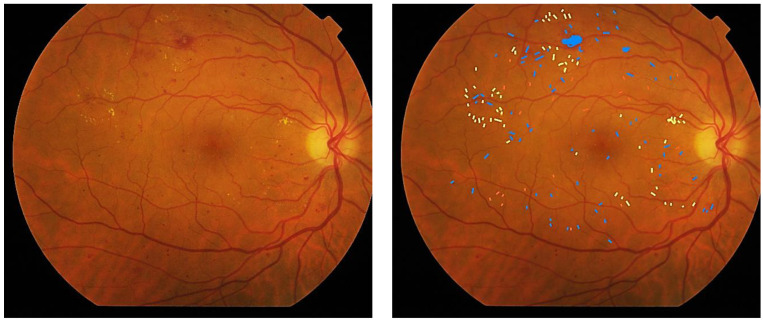
Example of a CFP image. (**Left**): original image. (**Right**): edited image highlighting MAs in red, Hmas in blue and HEs areas in yellow.

A total of 160 anonymized images of type 1 and 2 DM patients were analyzed and PDR patients were excluded: no RD (*n* = 30), mild NPDR (*n* = 30), moderate NPDR (*n* = 50) and severe NPDR (*n* = 50). The manual quantification of retinal lesions (MAs, Hmas and HEs) was conducted by two blinded, independent, certified graders from IOBA-UVA reading center who did not have any knowledge on the previous classification of the images. The quantification was performed only in the CFP NM-1 field. MAs were defined as round red lesions (RLs) with a well-defined edge and occasionally a brighter rim; Hmas were defined as RLs comprising round or irregularly shaped outlines of variable morphology (punctate, flame, and irregular edges) and HEs were identified following a protocol of morphological characteristics [31], being bright yellowish or white shinny flecks at different locations and with variable shapes and sizes. Figure 1 shows an example of a CFP image illustrating the presence of MAs, Hmas and HEs. 

Statistical analysis was performed using Graphpad Prism 8.0.1. Descriptive statistics, including number of values, mean, standard deviation (SD) and standard error of the mean (SEM) of each microvascular lesion for each DR degree level were calculated for all parameters. The intergroup (unpaired *T*-test) differences were performed (two-sided, α = 0.05)

## 3. Results

**Figure 2:**
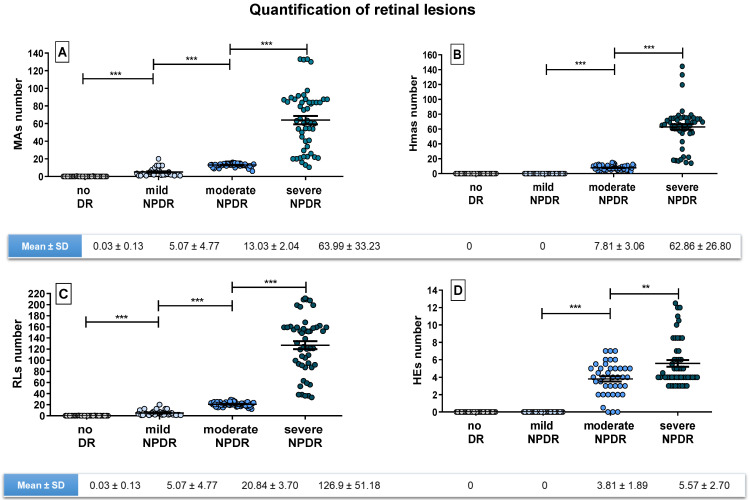
Quantification of a specific type of retinal lesions in a single central retinal field (NM-1), plotted against severity level (no DR, mild NPDR, moderate NPDR and severe NPDR. (**A**) Number of MAs quantified; (**B**) Number of Hmas quantified; (**C**) Number of RLs quantified, defined by the sum of MAs and Hmas; (**D**) Number of HEs quantified. Below each graph the mean ± SD is shown. Level of significance: *** *p* < 0.0001 and ** *p* < 0.001.

The number of MAs, Hmas, RLs and HEs quantified in the central NM-1 field were obtained by two independent, blinded graders who performed the quantification with no information on the severity level. After the counting process, each type of lesion was plotted separately against each DR severity level (no DR, mild, moderate and severe NPDR), as shown in Figure 2.

According to Figure 2, the number of MAs of patients with no DR was negligible, while mean MAs numbers in mild, moderate and severe NPDR patients were 5.07 ± 4.77, 13.03 ± 2.04 and 63.99 ± 33.23, respectively. According to these results, the mean MAs number significantly increased with the DR severity stage (no DR < mild DR < moderate DR < severe DR; *p* < 0.0001). 

Regarding Hmas, they were detected in moderate and severe NPDR patients and quantified as 7.81 ± 3.06 for moderate patients and 62.86 ± 26.80 for severe cases. 

RLs, representing the combination of both MAs and Hmas, showed the same trend as MAs quantification alone, but with higher numbers due to the influence of Hmas (0.03 ± 0.13 for no DR; 5.07 ± 4.77 for mild; 20.84 ± 3.7 for moderate and 126.9 ± 51.18 for severe NPDR). 

Finally, as expected, no HEs were identified in patients with no DR or mild NPDR, while the values increased to 3.81 ± 1.89 for moderate cases and 5.57 ± 2.70 for severe ones. The difference on the quantification of retinal lesions between severity levels was statistically significant in all cases. It is interesting to highlight the dispersion of HEs data compared to the lesions of vascular origin in NPDR patients. Such variability can be explained by the challenge for identifying these lesions, which usually form isolated plaques. In fact, a new method to detect them has been proposed based on the area they occupy rather than their amount [32].

**Figure 3:**
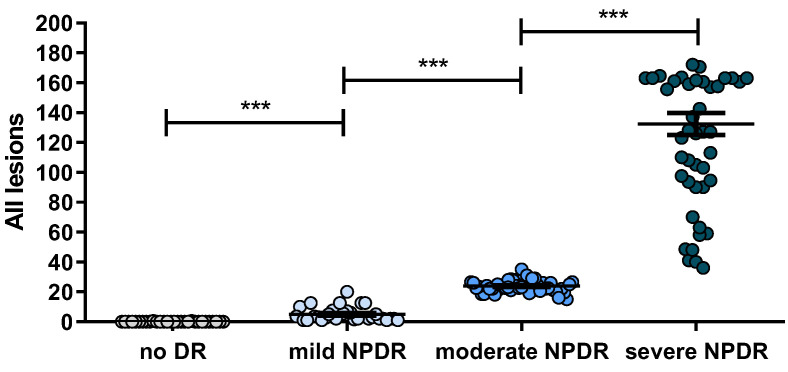
Quantification of all lesions in a single macula-centered NM-1 field, plotted against severity level (no DR, mild NPDR, moderate NPDR and severe NPDR. Level of significance: *** *p*< 0.0001.

In addition to considering the lesions separately, we also analyzed the quantifications by combining all types of lesions (MAs, Hmas, and HEs) in the same plot, as shown in Figure 3. 

The results obtained as the global microvascular picture considering the lesions altogether (Figure 3) indicate that their appearance in a single NM-1 central field significantly increases with DR severity.

## 4. Discussion

The results of the quantification of MAs, Hmas, RLs and HEs in a single NM-1 field revealed an increasing trend as DR severity level progresses, as expected. Of note is that the differences between the number of lesions counted among different severity degrees were statistically significant, suggesting that the detailed analysis of only one single NM-1 field could provide valuable information on the severity level. The clinical relevance of the central retinal field has been recognized in NPDR and DME, since the appearance of microvascular lesions close to the macula are closely associated with visual impairment and disease progression [33]. 

The results obtained in the present work bring to the consideration that, as MAs are the first observable hallmark of DR and their presence is differential across the severity stages, they could be proposed as a potential marker for assessing DR level and disease progression [34]. In fact, Vujosevic et al. [35] reported an early decrease in the number of MAs in the superficial capillary plexus (SCP) after 3 months treatment with sub-threshold micropulse laser and in the deep capillary plexus (DCP) after 6 months, in DME patients. The authors suggest that an evaluation of specific parameters in the SCP may help in determining the response of a treatment, supporting the role of microvascular parameters in disease evolution.

Interestingly, the quantitative ultra-widefield (UWF) fluorescein angiographic metrics reported by Ehlers et al. also concluded that MAs quantification, panretinal in that case, is also associated with DR severity [36]. Fluorescein Angiography (FA) is a valuable tool for quantifying MAs and differentiating them from Hmas, but its implementation for automated follow-up in the ophthalmologic practice is not foreseeable due to its invasiveness [37]. Along this line, different computer-aided methods for DR diagnosis have been developed based on fundus images to avoid the disadvantages of FA, demonstrating the potential and exploitability of this common ophthalmological technique. For instance, Wan et al. reported a novel convolutional neural network for lesion segmentation (MAs, Hmas, HEs and soft exudates) and showed high sensitivity, specificity and accuracy [38], while Hervella et al. proposed a deep learning methodology for improving the detection of MAs to achieve an early and accurate diagnosis of DR, also based on retinographies [39]. 

The relationship between microvascular abnormalities and disease severity has been reported by other authors, supporting our findings. In this regard, the work published by Xu et al. highlighted the correlation between the number of hemorrhagic lesions and the DR severity in both mild and moderate NPDR patients, based on the automated quantification of Hmas in CFP images [40]. 

Recent studies have also drawn the attention to other microvascular abnormalities, such as vessel density and the foveal avascular zone (FAZ), as potential and unbiased tools to quantitatively monitor DR or DME progression via optical coherence tomography angiography (OCTA) [16,41,42]. Interestingly, the studies led by Karasu et al. [41] and AttaAllah et al. [42] pointed out vessel density as a key indicator for disease progression in DME and macular perfusion. Although OCTA is indeed an appealing technique, we emphasize on finding a simplified approach based on CFP images, since this technique is the most widely used and affordable by current eyecare systems. 

While the development of new AI or deep learning algorithms for automatic image analysis aiming to differentiate MAs and Hmas is ongoing [28], their manual quantification is still challenging. In fact, the distinction between both lesions in CFP images is complicated and even impossible in many cases. A plausible solution to this issue, could be reporting MAs and Hmas together as red lesions (RLs), becoming a potential metrical approach for disease characterization. In fact, according to our results, the number of RLs is also related to the severity level with statistical significance. In the daily practice where fundus images are used very commonly to evaluate the posterior pole of the patients, the distinction of MAs from Hmas can be easily misleading. Therefore, even if detection errors are made, the association of both variables is still robust, which is clearly an added value at the clinical level. 

Although this work presents the potential of the quantification of specific retinal lesions in a single retinal NM-1 field to assess the level of DR severity, it has also limitations due to the relevance of peripheral fields in many cases of DR. Li et al. demonstrate that the typical clinical signs of DR, including MA, IRMA, capillary non-perfusion areas and NV, are commonly distributed in the inferior nasal mid-peripheral areas besides the posterior pole, assessed via fluorescein angiography and CFP [43]. This means that the microvasculature along retinal periphery needs to be adequately considered in order to have a more accurate DR evaluation. Fortunately, retinal photography and imaging has progressed substantially over the past decade, so new commercially available UWF systems have been developed, allowing imaging 200° of the retina in a single image, contrasting the 30° or 45° achieved in the ETDRS standard protocol or the JVN NM-I system [37].

In this line, Silva et al. report that UWF imaging is able to reveal substantially more diabetic retinal vascular pathology, even without the use of fluorescein angiography. In fact, the authors show that one third of Hmas/MAs, IRMA and NV are located predominantly outside the 7-fields ETDRS, suggesting that the severity level can be misleading [44]. In another study, Sears et al. highlighted the benefit of objective quantification for DR assessment for a more precise DR scoring system. The authors evaluated retinal lesions in UWF images based on subjective (performed by two masked graders) and quantitative assessments (lesion frequencies and surface area) and revealed that 22% of the identified lesions are distributed outside the ETDRS fields [45]. In the same way, Sadda et al. show that patients who have undergone PDR in 4 years are prone to showing more retinal lesions at the peripheral level [46].

Although the present work lacks the possibility to evaluate the periphery of the retina, the acquisition of a single central CFP field (NM-1) could be of high clinical value for a fast assessment of DR based on quantifiable variables. In fact, the use of numerical metrics over semi-quantitative or qualitative parameters could potentially allow the detection of observable improvements within the same level of severity, which, for instance, can be useful for evaluating the effectiveness of treatments. Moreover, the evaluation of only the central NM-1 field provides differential information on the DR severity level, avoiding the need to acquire multiple retail fields for the same purpose. This achievement is highly interesting for the current eyecare routine, given the cost- and resource-constrained healthcare systems. Finally, this affordable method has the advantage that the vast majority of ophthalmological settings have standard fundus cameras [47], while not all of them are equipped with UWF imaging, or the FA option is not feasible in routine eyecare visits. 

Overall, a standard simplified protocol for the daily practice should be agreed and deployed in the clinical health system based on CFP. The development of such clinical guideline, however, would be challenging due to different factors, starting from the choice of mydriatic (affording higher resolution images) versus non mydriatic cameras (a better option to avoid increase in intraocular pressure), the selection of the most suitable retinal field, and according to which system (7-field ETDRS, 3-fields JVN, etc.), among others.

This leads to the conclusion of Solomon et al. and Simó et al. [10,11] on the need for international ophthalmology leaders to systematically address how to quantify DR disease progression and to define the best techniques and protocols. In this sense, our goal is to make our results available to experts for their consideration as exploitable and quantifiable variables for DR detection and decision making based on microvascular counting of MAs and Hmas. 

## 5. Conclusions

As far as we are concerned, the present work is the first study that shows a statistically significant difference in the number of MAs, Hmas and RLs between DR severity levels quantified only in the central CFP field. Although it requires further validation, the quantification of lesions performed only in the central field and the DR severity level are closely and robustly interrelated. 

Furthermore, the potential of MAs and Hmas as quantifiable variables in fundus images for the disease severity level could be proposed as clinically relevant efficacy endpoints for clinical trials. 

In conclusion, our results could open up new tools to be implemented in the ophthalmological daily practice for recording the pathway of the disease and also for evaluating new therapeutic avenues for treating DR.

## Figures and Tables

**Table 1:** Comparison between International Clinical Diabetic Retinopathy (ICDR) and ETDRS severity scale level (adapted from [24].)

ICDR Disease Severity Level	Findings Observable upon Dilated Ophthalmoscopy	ETDRS Severity Scale Level
No apparent DR	No abnormalities	10—DR absent
Mild NPDR	Microaneurysms only	20—Microaneurysms only
Moderate NPDR	More than just microaneurysms but less severe NPDR	35—Mild NPDR 43—Moderate NPDR [The risk of subsequent development of PDR or CSME * is appreciable from this level] 47—Moderately severe NPDR
Severe NPDR	Any of the following and no signs of PDR – More than 20 intraretinal hemorrhages in each of four quadrants – Definite venous beading in two or more quadrants – Prominent IRMA ^†^ in one or more quadrants	53—Severe NPDR
PDR	One of both of the following: – Neovascularization – Vitreous/preretinal hemorrhage	61—Mild PDR 65—Moderate PDR 75—High-risk PDR 81, 85—Advanced PDR

* Clinically significant macular edema; † Intraretinal microvascular abnormalities.

## Data Availability

CFP images belong to the IOBA-UVA reading center data set from the DR blindness prevention program of Junta de Castilla y León (Regional Government). Data are not publicly available due to privacy restrictions.
